# Relative Age Effect in Swedish Male and Female Tennis Players Born in 1998–2001

**DOI:** 10.3390/sports6020038

**Published:** 2018-04-23

**Authors:** Göran Gerdin, Marie Hedberg, Carl-Axel Hageskog

**Affiliations:** Department of Sport Science, Linnaeus University, 351 95 Växjö, Sweden; marie.hedberg@lnu.se (M.H.); carl-axel.hageskog@lnu.se (C.-A.H.)

**Keywords:** relative age effect, tennis, Sweden, coaching, gender

## Abstract

The relative age effect (RAE) has been extensively debated and researched in both popular media and academic discourse. This study examined RAE in Swedish tennis players born in 1998–2001. The study was conducted in 2015–2016 and includes all ranked Swedish tennis players (*n* = 1835) registered in the Swedish Tennis Association database from the year 2014. The results show that when the birth dates of the corresponding Swedish population and all the ranked players are compared, they show a moderate RAE; however, the higher up they are in the ranking system, the greater the RAE becomes. Top 10 players display an average of 64.1% being born in the first half of the year. Some gender differences were also found, with a greater proportion of both higher and lower ranked females being born in the first half of the year. In our discussion of the findings we raise several issues that need to be addressed to provide more equal opportunities for all junior players regardless of birth date. Resolving ongoing problems associated with RAE in competitive sports such as tennis is important both in term of prolonged participation in the sport and increased performance. Suggestions made in this article include recognising RAE when designing the format of competitions/tournaments, not using official rankings until the juniors get older, addressing RAE in a “gender sensitive” way, and conducting further in-depth studies in which RAE is understood/examined as being associated with environmental factors. Although these findings show the RAE effect in Swedish tennis players, thus pointing at the need for further consideration in terms of ranking and selection procedures to ensure equal opportunities for player development, the study also concludes by reasserting an emphasis on a holistic approach to player development in which coaches focus on the developmentally appropriate needs and potential of each individual player regardless of their biological age.

## 1. Introduction

The topic of Relative Age Effect (RAE) has been a focus within popular books [1,2] and has garnered considerable attention among scientists and laypeople alike. Recently, there has been a growing interest in RAE in the sports context [3]. This phenomenon consists of a biased distribution of athletes’ birthdates. What is generally noticed is an overrepresentation of athletes born at the beginning of the competitive year, but an underrepresentation of people born at the end of it. Some authors have suggested that this effect is discriminatory for athletes born late in the competitive year [4]. Specifically, in the sport of tennis Edgar and O’Donoghue [5] underline the potential gains (e.g., status, fame, monetary) for a tennis player and argue the importance of every young player having an equal opportunity to excel as a tennis player regardless of their birth date.

This paper reports on findings from a larger study conducted in collaboration with the Swedish Tennis Association (STA) as part of their investigation into how they can provide for, facilitate, and encourage more young players in Sweden to practice, compete, improve, and become more successful in tennis and remain in the sport for longer. Part of this study sets out to examine how the birth date of a player influences their ongoing participation, performance, and success within competitive tennis in Sweden. The focus in this article is thus on understanding how RAE influences participation-, developmental- and competition-related outcomes [6] in the sport of tennis in Sweden.

In Sweden, like many other countries around the world, most national sporting associations have systems for ranking and selection in which they attempt to rank/identify young talented athletes who are then expected to be the most successful as young adults/adults (or seniors) [7]. The problem with these systems is that this ranking and selection occurs at an age when youth athletes are physically and mentally developing at different rates. To be physically able and strong is important in most sports [8], but often this is not the crucial factor for determining who will be the most successful. There are many other factors that need to be accounted for. The danger with these systems of ranking and selection thus is that many young athletes drop out of their sport, since they do not get the opportunity or encouragement that they should.

RAE is a term both within research on schooling and sports. In schooling it shows that the cut-off date for a year level plays a role, since those pupils that are relatively older than their peers have an advantage both in physical and cognitive areas. A significant amount of longstanding research on schooling [9,10], therefore, unsurprisingly, highlights inequalities in terms of relatively younger pupils having greater learning and achievement difficulties in schooling compared to their older peers. In sports with different age groups, the cut-off date is often the 1st of January, which means that those who were born in the first half of the year are privileged over those who were born later in the year. This privilege is seen when selections are made and in results from competitions in which the young athletes born earlier in the year being overrepresented. Most studies to date have shown that when the physical capabilities are important, as in sporting activities that are based on strength (e.g., sprint, throwing and jumping) and physical contact, there is a strong correlation between birth date and selection for teams/competitions. However, relatively few studies have been conducted in less physical contexts, and there is a growing amount of research showing that RAE also exists in less physically demanding sports such as chess [11], suggesting that this phenomenon is also more experience/psychology/socially determined than previously suggested.

Indeed, success cannot only be explained based on physical differences. Sport psychologist Harter [12] claims using theories about motivation that those young athletes who feel able/competent in their sport experience a higher degree of motivation than those who feel that they cannot develop any further. When young athletes feel acknowledged/recognised for their abilities/skills through results or selections they experience an increased sense of competence, ability, and, indeed, motivation compared to those who do not perform well or end up being selected. Different forms and formats of competition and explicit/transparent criteria for selection therefore become extra important at these early stages/ages. In addition, the surrounding peoples’ (e.g., parents, coaches, teachers) assessment and expectations of the young athletes can also create an advantage for those who early on are seen and categorised as ‘talented’ through the so-called Pygmalion- or Rosenthal effect [13,14]. For instance, a ranking system that from a young age favours those born early in the year could lead to higher expectations, aspirations, and ultimately success for those who are relatively older compared to the relatively younger athletes.

Previous research shows that there are certain sports with a pronounced RAE but also other sports where no significant RAE exists. These studies have, for instance, examined sports such as baseball, basketball, cricket, soccer, American football, gymnastics, handball, ice hockey, swimming, tennis, and volleyball. With team sports such as soccer and ice hockey in physical capability is important both in terms of technique and physical contact, there is an advantage for those who are born early in the year and, hence, are more likely to have developed further physically. It is important to note, however, that both growth and physical development during adolescence are age-influenced, but not necessarily age-dependent [15]. The timing and magnitude of changes during this time is regulated by both genetic and environmental factors that stimulate hormonal changes within the body [16]. Physical/biological maturation should therefore not be conflated with biological age, since it is also possible for some athletes that are relatively older to be delayed in biological maturation, as this will also be determined by a combination of genetic and environmental factors. Likewise, a relatively younger athlete can also be more advanced in biological maturation and experience physical advantages due to being an early developer.

In some studies [17], a significant RAE is observed in young athletes and also remains through to senior national team level. Other studies [3] show that this RAE decreases or even disappears over time when those young athletes who early on were a bit behind catch up. These studies demonstrate the importance of coaches and other people in the young athlete’s surroundings continuing to support and encourage them to keep practicing and competing despite not being acknowledged by, for instance, being selected for teams/competitions. In some sports, such as gymnastics and table tennis, there is instead a reversed RAE. In these sports that require coordination and fine motor skills over physical capability, there is an advantage of being smaller and lighter. Particularly in gymnastics, in which the movements/activities/events require the athlete to carry their own bodyweight and there are a lot of rotations, (a smaller) body size is a vital factor for success. Moreover, studies done on cricket, ice hockey, and baseball show RAE in positions that require physical strength. For example, there is strong RAE for the physically most demanding position in ice hockey (the goalie), with over two thirds of these athletes being born in the first half of the year [18].

In addition, in some sports, the nature of the tasks creates the opportunity to obtain a tactical advantage depending on whether an athlete is right- or left-handed (i.e., laterality), and this characteristic can interact with relative age and ultimately modify the influence of relative age [19]. For example, due to the lower frequency of left-handed players in the population, tennis players are more accustomed to playing right-handed players. Simply, opponents may be less perceptually accustomed to rapidly responding to the movement patterns, tactics, and orientation of left-handed players [20]. The result is that being left-handed can be a significant advantage in sports such as tennis. In fact, the advantage of handedness overrides the advantage of being relatively older. Loffing et al. [19] observed no RAE among left-handed tennis players but noted a significant RAE among right-handed players.

Musch and Grondin’s [3] comprehensive review of RAE across different sports showed that in North American ice hockey there today exists a distinct distribution with more players being born earlier in the year, while in the 1960s and 1970s no RAE was reported. However, in the Stanley Cup playoffs there is no difference between those players involved in the winning team compared to the average player in the league. In Germany, research on soccer has identified a significant RAE among youth soccer teams but also that this uneven birth date distribution has been evened out by the senior team stage. In the youth soccer teams (ages 18–22), 68% were born in the first half of the year, whereas in the top senior league (Bundesliga) the number was 49%. In swimming, there is RAE to some extent across different strokes, and in volleyball and handball there are RAEs for different playing positions.

Across different sports, it also varies between girls and boys and depending on the nature of the performance in various events. In tennis, it has been demonstrated that there is an RAE for British elite juniors [21] and Dutch juniors aged 12–16 [17]. More recently, Ulbricht et al. [22] showed that RAE is present in the selection of tennis juniors with the German Tennis Federation (DTB) competitive system and is more pronounced at higher competitive levels.

Moreover, in sports in which there is a high level of competition, there seems to be a greater chance of RAE. For the most popular game worldwide, soccer/football, in which the level of competition is very high in most countries/areas of the world, there is a significant RAE especially in the younger ages [23].

Research on schooling also suggests that there is RAE in relation to cognitive ability. The relatively older pupils have an advantage compared to the younger pupils, but this advantage/difference also seems to fade as time goes by. Also, it has been shown that those who have to struggle more at a younger age become more successful later in life, as motivation and aspiration are important factors for success [24]. These researchers argue that this is because schooling is compulsory and that those pupils who are/fall behind get extra help. Since sport is done on a voluntary basis, there is not the same room for and focus on helping everyone achieve/succeed, and those who are not improving or performing therefore end up dropping out [25].

A range of different attempts to diminish or resolve RAE have been made in which focus mainly has been on how age groups for practice and competition in sport are determined. The most common way of dividing athletes is to practice and compete with people who were born in the same calendar year, 1st of January to 31st of December. Efforts have been made to use revolving cut off dates and thus a different duration of age groups. Some researchers proposed the use of 15-month and 21-month age group period as a way of dealing with RAE [26]. Another suggestion was to use shorter age group periods, based on 9 rather than 12 months [27]. In swimming, attempts have been made to use individual cut off dates for different competitions in which the organisers themselves decide. Yet, other attempts have revolved around using different cut off dates for different sports, and in this way, give young athletes new opportunities in different sports. 

Another solution to the RAE problem has been to scan the young athletes for their “biological maturity age”. One study identified a range of 7–14 in “skeletal age” for a group of 11–12-year-old ice hockey players [28]. In martial arts sports, dividing athletes according to weight is commonly used. Dividing athletes according to ability/skill level is another common way of organising groups for practice and competition in sports.

None of the attempts described above to resolve the existence of RAE as associated with a loss of potential talent has been fully successful, since eliminating these effects has proven challenging, and different forms of “injustices” still exist in sports [3].

For instance, the attempts to shift the cut-off date and thereby reduce RAE have shown a shift in the location of the problem rather than providing a solution to it [4]. Attempts to scan for biological age raise important ethical questions; the problem of conflating relative age with physical/biological maturation [15] and the use of the ability/skill level grouping often result in young athletes feeling lost and disengaged when they have to move between different groups [29,30].

The aim of this study was to determine whether there exists any RAE in male and female junior tennis players in Sweden born between 1998 and 2001. A further aim was to see if any differences could be identified between male and female players.

## 2. Materials and Methods

To assess the prevalence of RAEs among Swedish junior tennis players, a substantial data set had to be collected from different sources. The existence of an RAE is generally determined by testing the statistical significance of the difference between the observed distribution of birth dates (in the sample) and the expected theoretical distribution (in the parent population) [31]. The data sources that were used in this study involved databases from the Swedish Tennis Association for all ranked tennis players born in 1998–2001 during the year 2014 (sample) and the Statistics Sweden [32] database for birthdates’ distribution of the Swedish population during the 1998–2001 period (parent population).

Among the sample, various subgroups were subsequently made and retained for further analyses. The first subgroup, defined as “ranked” players, included all male and female players born between 1998 and 2001 (13 to 16 years old) with an official ranking in the STA database (*n* = 1835). The two other subgroups were defined as “top 50” (*n* = 400) and “top 10” (*n* = 80) players.

To determine the existence of RAEs, player birth dates were firstly recorded to reflect their birth quartile (Q), according to the dates used for creating annual age groups. The cut-off date for the ranking of players in Swedish tennis is January 1st, and participants were divided into one of four groups. Therefore, Q1 = players born in January, February, and March; Q2 = players born April, May, and June; Q3 = players born in July, August, and September; and Q4 = players born in October, November, and December. It should be noted, however, that this way of categorising the players across four quartiles has its limitations. For example, players born on the 1st of January and 30th of March are considered the same, whereas the latter player and a player born on the 1st of April are considered distinct. Future studies could fruitfully express relative age as a decimal ranging from 0.00 (youngest) to 0.99 (the oldest), thus improving the quality and sensitivity of the data collected and analysed.

Chi-square tests were used to test the observed and expected birth distribution across the sample of players. Traditionally, the existence of RAE is determined by testing whether there is a statistically significant difference between the theoretical expected distribution of athletes’ dates of birth per quarter and the observed distribution using a chi-squared goodness-of-fit test [33]. The calculation of the theoretical expected distribution varies across different studies, in which some studies merely assume an even distribution of births among the four different quartiles [5,34]. However, the distribution of births in the general population is not uniform throughout the year. Concerns about these issues, and the potential for uncontrolled Type I error in large samples, have prompted the suggestion that actual population distributions need to be used as the comparator to relative age distributions of study samples [31].

When analysing the RAE, Delorme and Raspaud therefore claim that the actual frequencies of the parent population should be calculated as the expected theoretical distribution used in the chi-squared test to provide a more accurate measure of this discriminatory phenomenon. Hence, this study, as described above, accessed the Swedish data for birth statistics for all males and females born between 1998 and 2001 and used this data as the expected theoretical distribution for the parent population.

Pearson Chi-Squared tests were used to examine differences between boys and girls, and differences in rankings and participation levels. All calculations were performed using Microsoft Excel and SPSS (version 20.0, SPSS Inc., Chicago, IL, USA), and the level of significance was set at *p* < 0.05.

## 3. Results

**Figure 1:**
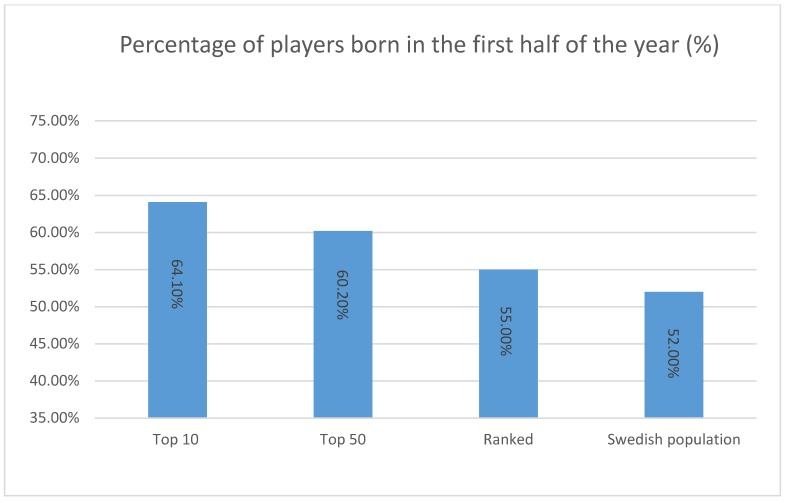
Representation of the different populations analysed (tennis players and Swedish population) born in the first half of the year.

The distribution of birth dates in the analysed tennis players, as well as the corresponding Swedish population, is shown in Figure 1 and Table 1.

Figure 1 shows the percentage of Swedish junior tennis players (males and females) with different playing statuses born in the first half of the year, and the birth distribution of the Swedish population born during 1998–2001. There is a somewhat balanced distribution of the Swedish population (52% born in the first half of the year (1stHY) and 48% born in the second half of the year (2ndHY), respectively). A moderate bias toward the 1stHY was observed for “ranked players” (55% and 45% for the 1stHY vs. the 2ndHY, respectively). On the contrary, 60.2% of players of the “top 50 players” were born in the first half (1stHY) of the year, and 39.8% in the second half (2ndHY). For the “top 10 players”, the birth months were even more skewed towards the 1stHY (64.1%) compared to the 2ndHY (35.9%) (Figure 1).

Overall, RAE appears to exist when the ranked male and female players are compared to the overall Swedish population and listed players. The RAE also seems to be greater higher up in the rankings.

When the corresponding Swedish population was used as the expected distribution (Table 1), results showed notable differences between birth quartiles, with more players born in the first quarters of the year. Although only statistical significance was observed for ranked (*p* = 0.013) and top 50 players (*p* = 0.031) born in 1999, and when all the age groups were pooled together (*p* = 0.002) along with a close-to-significant (*p* = 0.056) difference identified for top 50 players born in 2001, this needs to be interpreted in relation to the small sample size of this study, particularly for the top 10 player category.

The results presented in Table 1 also demonstrates that although the distribution of players born in the different quartiles varies somewhat between different age groups, the split between the first half and the second half of the year remains constant (65% 1stHY and 35% 2ndHY).

When all the players born between 1998 and 2001 are grouped together, it is made apparent how players born in the first quartile (Q1) have a distinct advantage over those born in the fourth quartile (Q4), particularly the higher up the rankings you look. For example, “top 10” and “top 50” players born in Q1 make up 33.8% and 32.5%, and those born in Q4 make up 17% and 13.7%, respectively.

Furthermore, when the male and female players were analysed separately, it was identified that RAE exists for both sexes but is greater for females (see Table 2 and Table 3). For both male and females, the higher up in the rankings, the greater the RAE is, except for boys born in 1998 and girls in 1999.

When all boys and girls born in 1998–2001 were pooled together separately, boys displayed being born in first half of the year ranging from 52.9% (ranked), 57% (top 50), and 62.5% for top 10, whereas same numbers for females were 56.1% (ranked), 63.5 (top 50), and 67.5% (top 10).

Statistical difference was only observed when all girls’ age groups were pooled together for ranked (*p* = 0.036) and top 50 players (*p* = 0.002), and close to statistical difference was observed for boys’ top 50 players born in 1999 (*p* = 0.079) and 2001 (*p* = 0.063), but this could again be explained by the small sample size.

Table 2 also shows how boys born in 1999 display a greater RAE compared to the other three age groups analysed, which involved as many as 68% top 50 and 70% top 10 players being born in the first half of the year. The girls displayed the same RAE and distribution for those born in 1998, although the other three girls’ age groups were not much different.

Being born in the fourth quartile seems to be a greater disadvantage for female compared to male players. When comparing all the male and female players, it is, for instance, worth noting that for males there is actually one more top 10 player born in the fourth quartile than the third (8 versus 7), whereas for girls the number is only 4 compared to 9 for the third quartile.

## 4. Discussion

The aims of the present study were to test the existence of RAEs in young Swedish male and female tennis players and to examine if these effects were influenced by age and/or skill level. A further aim was to investigate whether any differences exist in RAE between males and females.

The main findings of the present study were as follows: (1) an uneven birth distribution was present in Swedish junior competition tennis, (2) the observed effect was present in all of the age groups analysed and more pronounced higher up in the rankings, (3) the RAE was more significant for female players.

Firstly, the results presented above showed that RAE exists across all the age groups analysed (1835 players aged 13–16 years old) in the categories “ranked”, top “50”, and, in particular, the “top 10” players when compared to the corresponding Swedish population, with a greater percentage of players born in the first half of the year (Figure 1). Results are in line with previous research analysing the birth dates of tennis players (e.g., 12 to 18 years old), with players born in the 1stHY accounting for 60 to 86% of the whole population analysed [5,21,22].

When further analysing the possible age-related differences regarding RAEs, the findings revealed a skewed distribution of birth dates over the age groups analysed (males and female born in 1998–2001; Table 1) towards an earlier birth date. In contrast to previous studies, such as Ulbricht et al.’s [22] recent study on male German tennis players, this skewed distribution is evident and largely remains the same from a younger to an older age, with a relatively greater proportion of players born in the first quarters of the year. Thus, our results also show that the RAE in Swedish junior tennis players does in fact not seem to diminish as the athletes matures and gets older [35]; however, this could be due to the oldest age group analysed only being 16 at the time. Inclusion of older players could possibly have generated such data and needs to be considered in future studies examining RAE in Swedish tennis.

At the same time, this could add to previous findings that, compared to schooling and academic achievement, in which the advantage for relatively older pupils seems to fade, since schooling is compulsory and those who fall behind (e.g., relatively younger pupils) get extra support, sport is conducted on a voluntary basis and thus is not able to help those who are not succeeding or improving in the same way, and this results in more young athletes dropping out early [25]. This draws attention to the extra support that might be offered to relatively young athletes to prevent early dropping-out and promote development of all athletes regardless of birth date [29,30]. In terms of providing equal developmental opportunities for all, this could mean giving relatively younger athletes more time with the coach and playing time on the court/field.

When each age group was analysed separately, it was identified that RAE exist across all the age groups, and, more specifically, this is more pronounced higher up in the rankings. However, even at the “ranked” stage a moderate RAE seems to exist. Although the bias was less pronounced here (55.0%) than in the “top 50” (60.2%) and “top 10” (64.1%), we draw on Ulbricht et al.’s [22] speculation that among these ranked players, there is a process of “self-elimination” in the later born players, since the selection of this group is not based on ranking system, as in the top 50 and top 10 groups. Delorme, Boiche, and Raspaud [36] further claim that this form of self-elimination might be caused by later born players opting to drop out early in their competitive tennis career, since they likely experience more feelings of failure or inferiority compared to their peers who are born earlier in year and thus might have slight advantages in terms of physical, mental, and/or cognitive ability. These findings demonstrate the importance of giving extra support and encouragement to players born in the latter parts of the year right from the beginning (i.e., when they start playing competitive tennis). Competitions should therefore strive to design formats in an attempt to diminish RAE with the ambition of creating equal opportunities to experience success regardless of which part of the year the player was born. Indeed, in 2012 the International Tennis Federation (ITF) introduced the rule that all competitive tennis played for 10 years and under needs to use slower balls and/or modified court sizes. Similar initiatives might need to be put into place also for older juniors, ensuring equal opportunities for everyone to develop their tennis beyond the age of 10 years.

Furthermore, when looking higher up the rankings and across the four age groups analysed, top 50 players varied from 29% to 37% being born in the first quartile, whereas top 10 players ranged between 30% and 40%. In other words, the RAE identified at the entry (“ranked”) level increases as the competition gets stronger. These findings support previous research that has shown how the RAE is more likely to emerge at highly competitive tiers of participation than at recreational tiers [37]. In this study, the highest ranking tier examined was top 10; future studies might fruitfully include even higher tier grouping (e.g., top 5 or 3 players) to further strengthen this evidence of increased RAE higher up the ranking system. Since team selections for the national squads in tennis often are based on the top 3–5 players for each age group, this could be an important finding that would reassert the need and importance of recognising disadvantages and lost opportunities for players born in the latter quartiles of the year. In terms of team selection for the younger junior national teams, this could involve selecting players outside the very top ones, giving more players the experience of playing against other countries and thus building up a broader base of competitive players that potentially might lead to greater success for the older juniors and seniors. In addition, since absent or even reversed RAE has been found in recreational levels of different sports such as ice-hockey [38], it would be interesting to investigate if such a phenomenon also exists in recreational tennis in Sweden. 

The third main finding from this study relates to differences of the RAE observed between male and female junior tennis players. Although studies on RAE have been dominated by male populations, research suggests that sex is another significant individual constraint on the likelihood of RAE. Generally, RAEs have not been as consistently observed in female athlete populations, in which the size of the effects is smaller than among males. Male athletes have been seen as more susceptible to the relative age effect than women [39]. However, recent attempts to address the paucity of studies on female athletes have demonstrated that significant RAEs exist in women’s soccer [40], ice hockey [41], handball [42], and swimming [43,44]. Indeed, the results from this study add to these findings by highlighting RAE in junior female tennis players.

When all the different age groups analysed for girls were pooled together, girls being born in the first half of the year were 56.1% (“ranked”), 63.5 (“top 50”), and 67.5% (“top 10”) compared to the same sample of boys 52.9% (“ranked”), 57% (“top 50”), and 62.5% (“top 10”). This raises the issue of what makes the existence of RAE more prevalent in females compared to male competitive tennis in Sweden. One explanation might be found in the fact that females mature both physically and mentally at a more accelerated rate throughout their early teenage years, resulting in a more pronounced RAE at a younger age [45]. This gendered RAE phenomenon will have to be investigated further in future studies but still prompts the need to recognise that dealing with RAE in tennis, and indeed other sports, might have to be “sex/gender sensitive”. That is, different measures might need to be put into place to minimise the short- and long-term developmental consequences of RAE for female, as compared to male, junior tennis players. For instance, this could mean modifying the size of the courts and weight of the balls/racquets for younger female tennis juniors to lessen the physical advantage of those born earlier in the year, thus encouraging other important skills such as tactical awareness and sound technique. This finding could also add to the need to focus more on the differences in physical/biological maturation over relative/biological age, since physical development is influenced but not solely dependent on age [15]. Recognising that both girls and boys of the same biological age can vary greatly in physical, mental, and cognitive ability means acknowledging that a relatively older player can be disadvantaged, and a relatively younger player advantaged, due to being a late or early developer. A sole focus on relative/biological age when designing coaching and competition formats therefore risks creating new sporting injustices.

However, one age group of the boys (those born in 1999) displayed one of the highest RAEs in the whole sample studied, with 68% of the “top 50” and 70% “top 10” players being born in the first half of the year. A more in-depth study of this particular boys’ age group would be of interest, and, indeed, future studies may want to focus on such case studies in order to better our understanding of how particular constraints and environmental factors lead to RAE being more prominent in certain populations/age groups. This would also importantly further highlight the need to look at other factors together with birth dates such as social and environmental factors. As recently argued by Wattie, Schorer, and Baker [6], one of the difficulties in describing the relevance of the different constraints on RAEs is the obvious interaction between the different constraints. Young athletes only take on the fixed characteristic of a specific relative age once embedded in the context of school and/or youth sport and the environmental constraints therein (i.e., annual age grouping [3]). Thus, RAE can be seen to exist due to the interaction of individual (i.e., birth date) and environmental (i.e., annual age group) constraints [6].

Indeed, a recent study on “good sporting environments” in Sweden in which tennis was one of the sports included showed the complexity of the importance of environmental factors in nurturing and developing elite athletes [45]. A more in-depth study of “good” junior tennis development environments in Sweden is currently being conducted, which includes a focus on issues related to RAE for young male and female aspiring tennis juniors. Studies on RAE indicate that relatively older youth are simply advantaged by the fact that their individual constraints align with environmental constraints. That is, the individual constraints of relatively younger youth align with their ecology to promote positive developmental outcomes. The challenge for researchers and practitioners is to better align the individual constraints of relatively younger youth with environmental factors to optimize developmental plasticity. We therefore agree with Wattie et al. [6] who advocates that all youth, regardless of relative age, is imbued with the developmental characteristics of plasticity and the potential to develop positively.

To conclude, the results of the present study nonetheless may help improve the ranking policies and coaching of (elite) male and female junior tennis players in Sweden, facilitating the nurturing and better retainment of the number of players born in the latter part of the year. For instance, as a response to the findings in this project, the STA has already dropped rankings for juniors aged 12–14, and rankings are now only available from the year they turn 15. The aim is to minimise RAE in earlier years to encourage and include more juniors to stay longer in the sport, developing alongside and competing against juniors of different birth dates. Follow-up studies are planned to see whether any decreased RAE can be identified as a result of this initiative.

## Figures and Tables

**Table 1:** Season of birth distribution of young male and female tennis players and the corresponding Swedish population.

Age Group	Status	Q1 (%)	Q2 (%)	Q3 (%)	Q4 (%)	*n*	*p*
1998	Top 10	6 (30)	7 (35)	4 (20)	3 (15)	20	
	Top 50	31 (31)	30 (30)	21 (21)	18 (18)	100	
	Ranked	82 (25.7)	84 (26.3)	82 (25.7)	71 (22.2)	319	
	Sweden	22,438 (25.2)	23,751 (26.7)	23,522 (26.4)	19,317 (21.7)	89,028	
1999	Top 10	7 (35)	6 (30)	6 (30)	1 (5)	20	
	Top 50	33 (33)	33 (33)	22 (22)	12 (12)	100	0.031
	Ranked	107 (27.9)	117 (30.6)	100 (26.1)	59 (15.4)	383	0.013
	Sweden	22,249 (25.2)	23,776 (27)	22,634 (25.7)	19,514 (22.1)	88,173	
2000	Top 10	6 (30)	7 (35)	3 (15)	4 (20)	20	
	Top 50	29 (29)	27 (27)	27 (27)	17 (17)	100	
	Ranked	127 (24.2)	153 (29.0)	125 (23.7)	122 (23.1)	527	
	Sweden	22,748 (25.1)	23,932 (26.5)	23,258 (25.7)	20,503 (22.7)	90,441	
2001	Top 10	8 (40)	5 (25)	4 (20)	3 (15)	20	
	Top 50	37 (37)	21 (21)	21 (21)	21 (21)	100	0.056
	Ranked	173 (28.5)	154 (25.5)	140 (23.1)	139 (22.9)	606	
	Sweden	23,202 (25.3)	24,419 (26.7)	23,557 (25.7)	20,288 (22.2)	91,466	
All	Top 10	27 (33.8)	25 (31.3)	17 (21.2)	11 (13.7)	80	
	Top 50	130 (32.5)	111 (27.8)	91 (22.7)	68 (17)	400	0.002
	Ranked	489 (26.6)	508 (27.7)	447 (24.4)	391 (21.3)	1835	
	Sweden	90,682 (25.2)	95,878 (26.7)	92,971 (25.9)	79,619 (22.2)	35,9330	

**Table 2:** Birth distribution of young male tennis players and the corresponding Swedish population.

Age Group	Status	Q1 (%)	Q2 (%)	Q3 (%)	Q4 (%)	*n*	*p*
1998	Top 10	2 (20)	4 (40)	2 (20)	2 (20)	10	
	Top 50	14 (28)	13 (26)	9 (18)	14 (28)	50	
	Ranked	46 (23.7)	45 (23.3)	55 (28.4)	48 (24.7)	194	
	Sweden	11,560 (25.2)	12,221 (26.6)	11,988 (26.1)	10,171(22.1)	45,940	
1999	Top 10	4 (40)	3 (30)	2 (20)	1 (10)	10	
	Top 50	17 (34)	17 (34)	12 (24)	4 (8)	50	0.079
	Ranked	68 (28.2)	70 (29.1)	58 (24.2)	44 (18.5)	240	
	Sweden	11,414 (25.2)	12,332 (27.3)	11,617 (25.7)	9867 (21.8)	45,230	
2000	Top 10	3 (30)	3 (30)	2 (20)	2 (20)	10	
	Top 50	14 (28)	13 (26)	15 (30)	8 (16)	50	
	Ranked	77 (23)	93 (28)	83 (25)	80 (24)	333	
	Sweden	11,788 (25.3)	12,456 (26.7)	11,897 (25.5)	10,479 (22.5)	46,620	
2001	Top 10	4 (40)	2 (20)	1 (10)	3 (30)	10	
	Top 50	19 (38)	7 (14)	10 (20)	14 (28)	50	0.063
	Ranked	96 (26)	96 (26)	89 (24)	89 (24)	370	
	Sweden	12,086 (25.6)	12,519 (26.6)	12,030 (25.5)	10,503 (22.3)	47138	
All	Top 10	13 (32.5)	12 (30.0)	7 (17.5)	8 (20.0)	40	
	Top 50	64 (32.0)	50 (25.0)	46 (23.0)	40 (20.0)	200	
	Ranked	287 (25.2)	304 (26.7)	285 (25.1)	261 (23.0)	1137	
	Sweden	46,848 (25.3)	49,528 (26.8)	47,532 (25.7)	41,020 (22.2)	184,928	

**Table 3:** Birth distribution of young female tennis players and the corresponding Swedish population.

Age Group	Status	Q1 (%)	Q2 (%)	Q3 (%)	Q4 (%)	*n*	*p*
1998	Top 10	4 (40)	3 (30)	2 (20)	1 (10)	10	
	Top 50	17 (34)	17 (34)	12 (24)	4 (8)	50	
	Ranked	35 (28.2)	36 (29.1)	30 (24.2)	23 (18.5)	124	
	Sweden	10,878 (25.2)	11,530 (26.8)	11,534 (26.8)	9146 (21.2)	43,088	
1999	Top 10	3 (30)	3 (30)	4 (40)	0 (0)	10	
	Top 50	16 (32)	16 (32)	10 (20)	8 (6)	50	
	Ranked	44 (31)	33 (23.2)	38 (26.8)	27 (19)	142	
	Sweden	10,835 (25.2)	11,444 (26.6)	11,017 (25.7)	9647 (22.5)	42,943	
2000	Top 10	3 (30)	4 (40)	1 (10)	2 (20)	10	
	Top 50	15 (30)	14 (28)	12 (24)	9 (18)	50	
	Ranked	51 (26.4)	58 (30.0)	42 (21.8)	42 (21.8)	193	
	Sweden	10,960 (25.0)	11,476 (26.2)	11,361 (25.9)	10,024 (22.9)	43,821	
2001	Top 10	4 (40)	3 (30)	2 (20)	1 (10)	10	
	Top 50	19 (38)	14 (28)	11 (22)	6 (12)	50	
	Ranked	76 (32.3)	56 (23.8)	54 (23.0)	49 (20.9)	235	
	Sweden	11,116 (25.1)	11,900 (26.8)	11,527 (26.0)	9785 (22.1)	44,328	
All	Top 10	14 (35)	13 (32.5)	9 (22.5)	4 (10)	40	
	Top 50	67 (33.5)	61 (30.5)	45 (22.5)	27 (13.5)	200	0.002
	Ranked	206 (29.7)	183 (26.4)	164 (23.6)	141 (20.3)	694	0.036
	Sweden	43,789 (25.1)	46,350 (26.6)	45,439 (26.1)	38,599 (22.2)	174,177	
